# Asian Dust and Pediatric Emergency Department Visits Due to Bronchial Asthma and Respiratory Diseases in Nagasaki, Japan

**DOI:** 10.2188/jea.JE20150309

**Published:** 2016-11-05

**Authors:** Takahiro Nakamura, Masahiro Hashizume, Kayo Ueda, Atsushi Shimizu, Ayano Takeuchi, Tatsuhiko Kubo, Kunio Hashimoto, Hiroyuki Moriuchi, Hiroshi Odajima, Tasuku Kitajima, Kasumi Tashiro, Kunio Tomimasu, Yuji Nishiwaki

**Affiliations:** 1 Department of Environmental and Occupational Health, School of Medicine, Toho University, Tokyo, Japan; 1 東邦大学医学部社会医学講座衛生学分野; 2 Department of Paediatric Infectious Diseases, Institute of Tropical Medicine, Nagasaki University, Nagasaki, Japan; 2 長崎大学熱帯医学研究所小児感染症学分野; 3 Department of Environmental Engineering, Kyoto University Graduate School of Engineering, Kyoto, Japan; 3 京都大学大学院工学研究科 都市環境工学専攻 環境衛生学講座; 4 Regional Atmospheric Environment Section Center, National Institute for Environmental Studies, Tsukuba, Japan; 4 国立研究開発法人国立環境研究所地域環境研究センター; 5 Department of Preventive Medicine and Public Health, School of Medicine, Keio University, Tokyo, Japan; 5 慶應義塾大学医学部衛生学公衆衛生学教室; 6 Department of Public Health, School of Medicine, University of Occupational and Environmental Health, Kitakyushu, Fukuoka, Japan; 6 産業医科大学医学部公衆衛生学; 7 Department of Paediatrics, Graduate School of Biomedical Sciences, Nagasaki University, Nagasaki, Japan; 7 長崎大学大学院医歯薬学総合研究科 小児科学; 8 Department of Paediatrics, National Hospital Organization Fukuoka Hospital, Fukuoka, Japan; 8 国立病院機構福岡病院 小児科; 9 Department of Paediatrics, Kamigoto Hospital, Nagasaki, Japan; 9 長崎県上五島病院 小児科; 10 Department of Paediatrics, Isahaya General Hospital, Nagasaki, Japan; 10 独立行政法人地域医療機能推進機構諫早総合病院小児科; 11 Nagasaki Municipal Primary Emergency Medical Center, Nagasaki, Japan; 11 長崎市夜間急患センター

**Keywords:** Asian dust, emergency department visits, bronchial asthma, respiratory diseases, children, 黄砂, 救急受診, 気管支喘息, 呼吸器疾患, 小児

## Abstract

**Background:**

The adverse health effects of Asian dust (AD) on the respiratory system of children are unclear. We hypothesized that AD events may lead to increased visits by children to emergency medical centers due to bronchial asthma and respiratory diseases, including bronchial asthma.

**Methods:**

We used anonymized data on children receiving primary emergency treatment at Nagasaki Municipal Primary Emergency Medical Center, Japan between March 2010 and September 2013. We used Light Detection and Ranging (LIDAR) data to assess AD exposure and performed time-stratified case-crossover analyses to examine the association between AD exposure and emergency department visits. The main analysis was done with data collected from March through May each year.

**Results:**

The total number of emergency department visits during the study period was 756 for bronchial asthma and 5421 for respiratory diseases, and the number of “AD days” was 47. In school children, AD events at lag day 3 and lag day 4 were associated with increased emergency department visits due to bronchial asthma, with odds ratios of 1.837 (95% confidence interval [CI], 1.212–2.786) and 1.829 (95% CI, 1.179–2.806), respectively. AD events were significantly associated with respiratory diseases among preschool children at lag day 0, lag day 1, and lag day 2, with odds ratios of 1.244 (95% CI, 1.128–1.373), 1.314 (95% CI, 1.189–1.452), and 1.273 (95% CI, 1.152–1.408), respectively. These associations were also significant when the results were adjusted for meteorological variables and other air pollutants.

**Conclusions:**

The study findings suggested that AD exposure increases emergency department visits by children.

## Abstract (ja)

**【背景と目的】:**

黄砂曝露による小児の呼吸器疾患への影響は未だ十分に明らかでない。黄砂と小児気管支喘息やそれを含む呼吸器疾患による救急外来受診との関連について検討を行った。

**【方法】:**

長崎市夜間急患センターを受診した小児の匿名化データ(2010年3月～2013年9月)を用いた。黄砂曝露の定義はLIDAR（Light Detection and Ranging）観測のデータに基づいて行い黄砂と救急受診の関連はTime Stratifiedケースクロスオーバー解析を行い関連の強さはオッズ比と95%信頼区間(CI)により示した。主たる解析は黄砂が多く飛散する3月から5月に限定して行った。

**【結果】:**

対象期間の救急受診は気管支喘息756件とそれを含む呼吸器疾患5,421件で、黄砂日は47日であった。学童に関しては、非黄砂日に対して黄砂日から3日後と4日後の気管支喘息受診のオッズ比はそれぞれ1.837 (95%CI; 1.212-2.786)、1.829 (95%CI; 1.179-2.806)であった。一方で未就学児は黄砂日当日、翌日、2日後にかけて関連がみられ、オッズ比はそれぞれ1.244 (95%CI; 1.128-1.373)、1.314 (95%CI; 1.189-1.452)、1.273 (95%CI; 1.152-1.408)であった。これらの関連は気象因子や他の大気汚染物質で調整後も同様に統計学的に有意であった。

**【結論】:**

黄砂曝露により、気管支喘息や呼吸器疾患による小児救急受診が増加することが示唆された。

## INTRODUCTION

Asian dust (AD) is a natural phenomenon in which wind carries dust over large distances from the Yellow River basin and deserts in northern China and Mongolia. It is now regarded as a worldwide environmental problem caused by manmade deforestation, soil degradation, and desertification.^1^^–^^3^

AD reaches Japan throughout the year, but the amount increases from February and peaks in April.^4^ AD is a common problem throughout Northeast Asia, but the type and degree of damage it causes may vary, depending on the distance from the source of the dust.^1^ In Japan, AD most often affects western Japan. Nagasaki Municipal Primary Emergency Medical (NMPEM) Center is located in the center of the city and is the primary night-time and holiday emergency hospital for children living in the Nagasaki area. Children appear to be more vulnerable than adults, in terms of both susceptibility and response to environmental changes, including air pollution.^5^^–^^7^

Although studies on the effects of AD on the respiratory system in children have been carried out in Taiwan, China, and Korea,^8^^–^^13^ the results have been contradictory. Some studies reported an increased risk of clinic visits^8^ and hospitalization for asthma,^14^ whereas another study reported a negative association with emergency asthma hospitalization.^15^

We speculated that the effects of AD may vary between preschool and school children because of differences in their daily activities. However, there is very little evidence to support this. In addition, if AD does indeed affect the respiratory system of children, the dose-response relationship needs to be investigated. Recent epidemiological studies from Korea and Japan have measured levels of AD using Light Detection and Ranging (LIDAR).^14^^,^^16^^,^^17^ LIDAR is a type of radar that uses laser beams instead of radio waves. The laser beams emitted from the ground are scattered by fine particles in the air, and, by measuring the scattered laser light, operators can determine the vertical distribution of dust particle concentration and changes over time.^18^ Such measurements provide reliable quantitative data on AD levels in the atmosphere.

The objective of this study was to examine data from the NMPEM Center to identify any relationship between AD and emergency department visits by children.

## METHODS

### Study area

**Figure 1.**
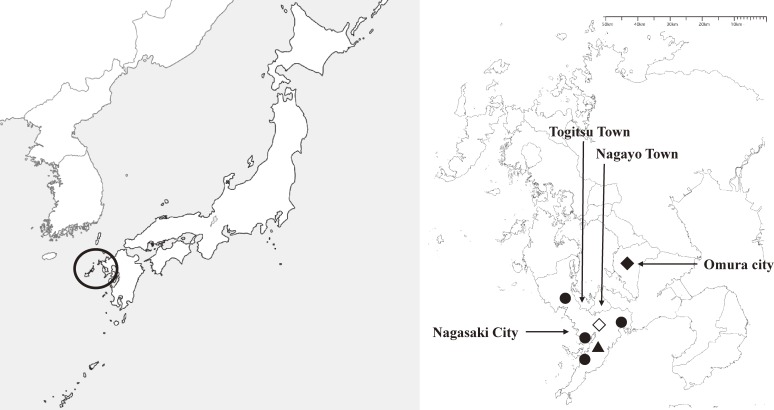
Map of Japan (left) and Nagasaki City (right) ○ shows Nagasaki Prefecture (left); ◇ shows Nagasaki Municipal Primary Emergency Medical Center (right); ◆ shows LIDAR in Omura City (right); ● shows four air pollution monitoring stations in Nagasaki city (right); ▲ shows Nagasaki Marine Observatory. LIDAR, light detection and ranging.

Nagasaki Prefecture borders Saga Prefecture to the east, but it is otherwise surrounded by the East China Sea (Figure 1). The prefecture’s capital is Nagasaki City. The NMPEM Center serves the whole of Nagasaki City and two neighboring towns (Togitsu and Nagayo in Nishisonogi District). In 2013, the total population of this area was approximately 500 000, with about 25 000 children aged 0–5 years and about 46 000 children aged 6–15 years.^19^ Approximately 90% of the patients who access the medical center live in the above-mentioned area, according to unpublished information from the center. The center offers emergency services for children 7 days per week from 20:00 to 06:00. Several pediatricians work in shifts to provide these services.

### Collection of data on emergency department visits

We obtained unlinkable anonymized data (research ID number date of visits, date of birth, diagnosis, and treatment) on all children aged 0–15 years who received primary emergency treatment at the Center between March 2010 and September 2013. We created two diagnostic categories: 1) bronchial asthma and 2) respiratory diseases, including bronchial asthma, bronchitis, pharyngitis, and common cold.

In order to focus on the effects of AD, we excluded patients diagnosed with specific respiratory pathogens, such as influenza viruses, respiratory syncytial virus, or *mycoplasma* infections.

### Measurement of AD

To assess exposure to AD during the study period, we used LIDAR data obtained from an installation in Omura City (Figure 1), which is approximately 30 km from Nagasaki City. LIDAR utilizes polarized laser light to recognize shape differences and can distinguish AD particles from other air pollutants, which are generally spherical. If the lower atmosphere is well mixed, the concentration of AD on the ground is similar to that between 120 m and 270 m above ground, the lowest level for which LIDAR data are available.^18^ The National Institute for Environmental Studies LIDAR network can estimate the dust extinction coefficients of non-spherical and spherical components.^20^ LIDAR measurements were compiled as total 24-hour data (midnight to midnight). AD extinction coefficients larger than 1/km and spherical extinction coefficients larger than 2/km were excluded, because these data may be influenced by meteorological factors, such as cloud, fog, rain, and snow.^20^

In our study, we defined “AD days” as when all of the following criteria were met^21^:

1. The daily maximum suspended particulate matter (SPM) concentration was over 50 µg/m^3^.
2. The daily maximum AD extinction coefficient as measured by LIDAR was more than 0.05/km.
3. The correlation coefficient between the hourly AD extinction coefficient and hourly SPM concentration was a fixed value.

For SPM concentrations, we used the daily average of hourly measurements made by four air pollution monitoring stations. SPM is defined under the Japanese Air Quality Standard as any particle with an upper 100% cut-off point of 10 µm in aerodynamic diameter.^22^

### Other air pollutants and meteorological data

Data on other air pollutants (sulfur dioxide [SO_2_], nitrogen dioxide [NO_2_], and photochemical oxidants [Ox]) were collected from four air pollution monitoring stations in Nagasaki City. Daily average concentrations of SO_2_ and NO_2_ were calculated from hourly concentrations measured at each station. Ox is defined as mixtures of ozone and other secondary oxidants generated by photochemical reactions and is considered to be a proxy for ozone. Maximum hourly concentrations of Ox measured at each station were used. Data based on fewer than 20 hourly measurements on any one day were excluded.^15^^,^^20^

Daily temperature and relative humidity data in Nagasaki City were obtained from the Nagasaki Marine Observatory of the Japanese Meteorological Agency. We calculated the daily average temperature and relative humidity from data collected hourly at Nagasaki Marine Observatory (between 00:00 and 23:00), and recorded the maximum and minimum temperatures.

### Statistical analysis

We used the Mann-Whitney U test or Student’s *t*-tests to compare AD extinction coefficients, spherical extinction coefficients, SPM, other air pollutants and meteorological data on AD days and non-AD days.

A time-stratified case-crossover analysis was used to examine the relationship between AD and emergency department visits due to bronchial asthma and respiratory diseases. In the same way as in a matched case-control analysis, this analysis assigned the day on which a patient visited the clinic with bronchial asthma or respiratory disease as the case day, and comparisons were made with control days chosen on the same day of the week earlier or later in the same month of the same year.^23^^,^^24^ The advantage of this design is that it controls for time-invariant personal factors, the effects of long-term trends, seasonality, and the day of the week. The strength of the association between AD days and emergency clinic visits was shown by the odds ratios (ORs) and 95% confidence intervals (CIs) of the conditional logistic models. *P* values of less than 0.05 were considered to indicate statistical significance.

Following the Japanese Pediatric Guideline for the Treatment and Management of Asthma 2012, we classified three age categories: under 2 years old, 2 through 5 years old, and 6 through 15 years old^25^; however, because we found that the results for the first two groups were similar, we combined them in the subsequent analysis. Thus, the preschool children group consisted of patients aged 0 through 5 years, and the school children group consisted of those aged 6 through 15 years. We analyzed combined data for boys and girls, because the associations between AD and emergency department visits did not differ by sex.

In addition to AD days, we added temperature and relative humidity as co-variables,^20^ along with SO_2_, NO_2_, and Ox concentrations, and the spherical extinction coefficients (these variables were average on the case day, lag day 1, and lag day 2). We constructed three adjusted models. In the basic model, we adjusted for temperature and relative humidity. In the single-pollutant model, we adjusted for one of the air pollutants (SO_2_, NO_2_, or Ox), in addition to the variables in the basic model. In the multi-pollutant model, we adjusted for two of the air pollutants, in addition to the variables in the basic model.

Because the effects of AD can persist over several days, we examined the effects with several lag times; the case day (lag day 0), lag day 1, lag day 2, lag day 3, lag day 4, and lag day 5 as a single lag model (the model included only one exposure variable). We also examined the lagged cumulative effects from lag day 0 to day 1 (designated lag 01), lag day 0 to day 2 (lag 02), lag day 0 to day 3 (lag 03), lag day 0 to day 4 (lag 04), and lag day 0 to day 5 (lag 05).

In the basic model, we used not only the binomial index of AD but also the AD extinction coefficients, with reference to the methods of Ueda et al.^26^ We classified results into three categories: Non-AD days (extinction coefficient <0.047/km), moderate AD days (extinction coefficient 0.047–0.065/km), and heavy AD days (extinction coefficient ≥0.066/km), according to the median (0.047/km) and 75th percentile (0.066/km) value of the AD extinction coefficients.

In addition, several sensitivity analyses were performed. First, because airway inflammation and sensitivity to environmental stimuli may persist after an initial attack, we carried out further analyses after excluding visits within 4 weeks of the first visit. Second, we conducted analyses using data for the whole year rather than just those for March through May.

All analyses were performed with STATA ver. 12.1 (StataCorp, College Station, TX, USA).

### Ethical considerations

This study was a retrospective observational study using unlinkable anonymized data. It was approved by the Ethics Committee of Nagasaki University Hospital (Approval No. 13082663) and Toho University School of Medicine (Approval No. 25100).

## RESULTS

### AD days

**Figure 2.**
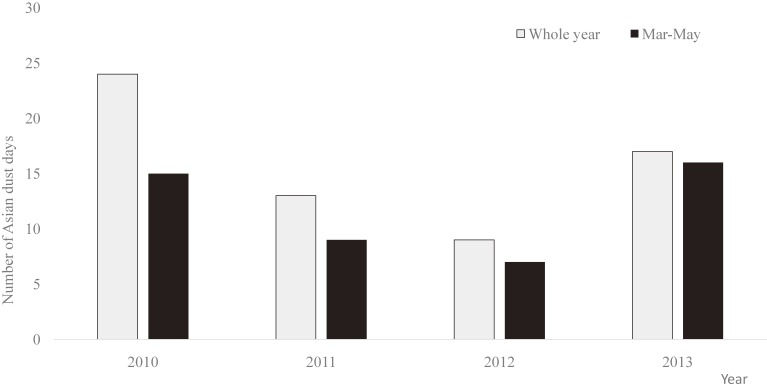
Number of Asian dust days from March 2010 through September 2013.

LIDAR measurements showed that the total number of AD days during the study period (March 2010 through September 2013) was 63 (Figure 2). The largest number of AD days was observed in 2010, and most (47/63) occurred in spring (March to May) each year, although they were also occasionally observed in November and December. Therefore, most of our analyses were based on data collected in March through May.

### Primary emergency department visits to Nagasaki Municipal Primary Emergency Medical Center

During the 3.5-year study period (March 2010 through September 2013) there were 43 892 emergency department visits, of which 34 170 were made by patients aged 15 years or below (18 805 boys and 15 365 girls). Of these, 9937 were made between March and May; 756 involved bronchial asthma (mean age of the patients, 3.78 years) and 5421 were due to respiratory diseases (mean age, 3.45 years) (Table 1). Boys and preschool children made up the majority in both diagnostic categories. The largest number of visits due to bronchial asthma occurred in May 2010, and the smallest in March 2012; May 2011 saw the largest number of visits due to respiratory diseases, and March 2010 saw the smallest.

**Table 1.** Number of emergency department visits (March to May)

		Bronchial asthma			Respiratory diseases		
	
		All Children 0–15 years old (*n* = 756)	Preschool Children 0–5 years old (*n* = 551)	School Children 6–15 years old (*n* = 205)	All Children 0–15 years old (*n* = 5421)	Preschool Children 0–5 years old (*n* = 4070)	School Children 6–15 years old (*n* = 1351)
Age, years	Mean	3.78	1.91	8.82	3.45	1.5	9.35
	Median	3	2	9	2	1	9

Sex	Male	474	337	137	2942	2198	744
	Female	282	214	68	2479	1872	607

Year	2010	214	159	55	1205	975	230
	2011	197	138	59	1485	1111	374
	2012	156	112	44	1343	956	387
	2013	189	142	47	1388	1028	360

Month	March	212	154	58	1626	1150	476
	April	250	182	68	1749	1340	409
	May	294	215	79	2046	1580	466

### AD, other air pollutants, and meteorological data

We compared the levels of AD, other air pollutants, and meteorological data on AD days and non-AD days from March through May, 2010–2013 (Table 2). The mean AD coefficient on AD days (0.07/km) was significantly higher than that on non-AD days (0.03/km). There were differences in the concentrations of other air pollutants and meteorological variables (except NO_2_ and relative humidity) on AD days and non-AD days.

**Table 2.** Summary of data on Asian dust, other air pollutants, and meteorological factors (March 2010–May 2013)

	Non-Asian-dust days						Asian-dust days						*P* value^a,b^
	
	Number of days	Mean	SD	25th percentile	Median	75th percentile	Number of days	Mean	SD	25th percentile	Median	75th percentile	
Asian dust extinction coefficients, /km	320	0.03	0.03	0.01	0.02	0.03	47	0.07	0.08	0.03	0.05	0.07	<0.01^a^

Spherical extinction coefficients, /km	320	0.13	0.11	0.05	0.10	0.16	47	0.19	0.13	0.09	0.16	0.24	<0.01^a^

SO_2_, ppb	321	1.52	0.77	1.00	1.30	1.80	47	1.98	0.97	1.20	1.60	2.50	<0.01^a^
NO_2_, ppb	321	7.58	2.28	6.00	7.40	9.00	47	8.11	2.60	6.60	7.70	9.40	0.25^a^
Ox, ppb	321	42.98	8.99	38.40	43.40	48.70	47	46.74	8.99	41.50	46.20	53.60	0.015^a^
SPM, µg/m^3^	321	23.95	11.74	16.00	21.60	29.30	47	53.14	33.39	34.30	44.60	56.80	<0.01^a^

Average Temperature, °C	321	14.78	4.4	11.5	14.9	18.6	47	17.4	3.9	15.4	18.0	20.3	<0.01^a^
Maximum Temperature, °C	321	18.88	4.7	15.3	19.1	22.6	47	22.1	4.2	19.5	22.4	25.6	<0.01^a^
Minimum Temperature, °C	321	11.13	4.7	7.5	10.8	15.0	47	13.1	4.2	10.5	13.9	16.6	<0.01^a^
Humidity, %	321	67.49	13.3	58.0	68.0	76.0	47	67.2	8.4	62.0	68.0	74.0	0.56^b^

SD, standard deviation; SPM, small particulate matter.

*P* value for ^a^Mann-Whitney U test and ^b^*t*-test.

### Association between AD and emergency department visits

**Figure 3.**
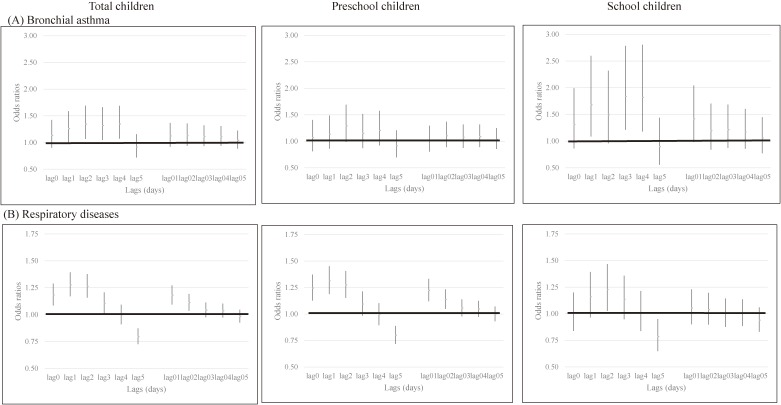
Crude associations between Asian dust and emergency department visits due to bronchial asthma (A) and respiratory diseases (B) by age group (vertical bar represents 95% confidence interval).

The crude ORs by age group for emergency department visits on AD days due to bronchial asthma and respiratory diseases are shown in Figure 3.

In all children, AD events at lag day 2, lag day 3, and lag day 4 were associated with increased emergency department visits due to bronchial asthma, with ORs of 1.343 (95% CI, 1.067–1.690), 1.318 (95% CI, 1.047–1.660), and 1.344 (95% CI, 1.071–1.687), respectively. The association was particularly prominent in school children, with ORs of 1.837 (95% CI, 1.212–2.786) for lag day 3 and 1.820 (95% CI, 1.179–2.808) for lag day 4. In preschool children, the crude ORs were around 1.1 for lag day 2 through lag day 5, without statistical significance.

As for respiratory diseases, AD events at lag day 0, lag day 1, and lag day 2 were significantly associated with emergency department visits by all children with ORs of 1.182 (95% CI, 1.084–1.288), 1.277 (95% CI, 1.169–1.393), and 1.262 (95% CI, 1.156–1.377), respectively. ORs for lag 01 and lag 02 were also increased. Unlike with bronchial asthma, however, the association was seemed stronger in preschool children than in school children, with ORs of 1.244 (95% CI, 1.128–1.373) for lag day 0, 1.314 (95% CI, 1.189–1.452) for lag day 1, and 1.273 (95% CI, 1.152–1.408) for lag day 2, respectively. In schoolchildren, there was no statistically significant association except for lag day 2 (crude OR 1.227; 95% CI, 1.026–1.467).

We further adjusted for meteorological data and other air pollutants in our basic, single-pollutant, and multi-pollutant models. We also adjusted for spherical extinction coefficients in addition to the covariates in the basic model. These adjustments did not change the results substantially. In Table 3, we present the adjusted ORs of the association between AD days and emergency department visits due to bronchial asthma in school children, because a statistically significant association was observed in the crude analysis. Likewise, we present the adjusted ORs of the association between AD days and visits due to respiratory diseases in preschool children in Table 4. Although adjustment for covariates slightly attenuated the ORs, the results were again similar to those obtained using the basic model.

**Table 3.** Association between Asian dust and emergency department visits by bronchial asthma among school children in the basic, single-pollutant, and multi-pollutant models

	Basic model	Single-pollutant model (Basic model+single-pollutant) Adding to basic model, one air pollutant was adjusted			Multi-pollutant model (Single-pollutant model+other pollutant) Adding to basic model, two air pollutants were adjusted			Adding to basic model
			
	Adjusted by temperature+humidity	SO_2_	NO_2_	Ox	SO_2_+NO_2_	SO_2_+Ox	NO_2_+Ox	Spherical Extinction Coefficients
	OR (95% CI)	OR (95% CI)	OR (95% CI)	OR (95% CI)	OR (95% CI)	OR (95% CI)	OR (95% CI)	OR (95% CI)
Lag 0	1.291 (0.850–1.961)	1.173 (0.760–1.811)	1.298 (0.854–1.974)	1.150 (0.738–1.790)	1.164 (0.754–1.798)	1.131 (0.725–1.765)	1.127 (0.724–1.757)	1.343 (0.867–2.081)
Lag 1	1.599 (1.032–2.478)	1.446 (0.911–2.294)	1.634 (1.048–2.546)	1.454 (0.916–2.307)	1.459 (0.918–2.316)	1.409 (0.883–2.250)	1.461 (0.921–2.317)	1.701 (1.080–2.681)
Lag 2	1.394 (0.891–2.182)	1.206 (0.740–1.964)	1.465 (0.915–2.344)	1.233 (0.765–1.990)	1.265 (0.770–2.080)	1.169 (0.714–1.914)	1.297 (0.798–2.110)	1.436 (0.898–2.298)
Lag 3	1.787 (1.174–2.721)	1.675 (1.088–2.579)	1.853 (1.205–2.848)	1.670 (1.080–2.581)	1.745 (1.127–2.701)	1.644 (1.061–2.547)	1.727 (1.113–2.680)	1.845 (1.204–2.826)
Lag 4	1.807 (1.163–2.809)	1.749 (1.121–2.730)	1.831 (1.175–2.852)	1.693 (1.076–2.666)	1.790 (1.145–2.799)	1.717 (1.088–2.710)	1.703 (1.082–2.680)	1.831 (1.173–2.857)
Lag 5	0.894 (0.550–1.453)	0.885 (0.542–1.442)	0.896 (0.551–1.458)	0.877 (0.538–1.430)	0.901 (0.552–1.469)	0.877 (0.538–1.432)	0.891 (0.547–1.451)	0.895 (0.550–1.457)

Lag 01	1.329 (0.915–1.930)	1.202 (0.809–1.786)	1.342 (0.922–1.955)	1.195 (0.800–1.787)	1.198 (0.805–1.781)	1.164 (0.775–1.748)	1.180 (0.789–1.765)	1.395 (0.947–2.056)
Lag 02	1.136 (0.794–1.625)	0.979 (0.659–1.454)	1.153 (0.800–1.663)	0.976 (0.655–1.455)	0.990 (0.666–1.471)	0.930 (0.617–1.401)	0.979 (0.656–1.459)	1.171 (0.804–1.705)
Lag 03	1.173 (0.840–1.639)	1.036 (0.718–1.485)	1.195 (0.848–1.684)	1.021 (0.699–1.491)	1.054 (0.730–1.523)	0.984 (0.668–1.450)	1.026 (0.702–1.500)	1.217 (0.858–1.726)
Lag 04	1.131 (0.822–1.557)	1.012 (0.716–1.431)	1.146 (0.828–1.587)	0.998 (0.699–1.423)	1.025 (0.725–1.449)	0.967 (0.672–1.389)	1.000 (0.701–1.426)	1.175 (0.844–1.635)
Lag 05	1.020 (0.739–1.408)	0.897 (0.631–1.276)	1.029 (0.741–1.429)	0.889 (0.623–1.269)	0.910 (0.640–1.295)	0.854 (0.592–1.231)	0.895 (0.627–1.278)	1.053 (0.756–1.467)

CI, confidence interval; OR, odds ratio.

**Table 4.** Association between Asian dust and emergency department visits by respiratory diseases among preschool children in the basic, single-pollutant, and multi-pollutant models

	Basic model	Single-pollutant model (Basic model+single pollutant) Adding to basic model, one air pollutant was adjusted			Multi-pollutant model (Single-pollutant model+other pollutant) Adding to basic model, two air pollutants were adjusted			Adding to basic model
			
	Adjusted by temperature+humidity	SO_2_	NO_2_	Ox	SO_2_+NO_2_	SO_2_+Ox	NO_2_+Ox	Spherical Extinction Coefficients
	OR (95% CI)	OR (95% CI)	OR (95% CI)	OR (95% CI)	OR (95% CI)	OR (95% CI)	OR (95% CI)	OR (95% CI)
Lag 0	1.245 (1.129–1.374)	1.217 (1.099–1.347)	1.255 (1.137–1.385)	1.256 (1.129–1.396)	1.216 (1.099–1.347)	1.242 (1.117–1.382)	1.250 (1.124–1.390)	1.335 (1.202–1.483)
Lag 1	1.312 (1.186–1.451)	1.284 (1.156–1.426)	1.334 (1.204–1.477)	1.337 (1.198–1.492)	1.295 (1.165–1.439)	1.320 (1.182–1.475)	1.339 (1.200–1.495)	1.420 (1.276–1.580)
Lag 2	1.272 (1.148–1.410)	1.242 (1.117–1.382)	1.310 (1.178–1.456)	1.281 (1.148–1.429)	1.273 (1.143–1.418)	1.264 (1.132–1.411)	1.301 (1.165–1.453)	1.335 (1.201–1.484)
Lag 3	1.090 (0.980–1.213)	1.069 (0.960–1.190)	1.103 (0.990–1.229)	1.080 (0.969–1.204)	1.085 (0.973–1.209)	1.072 (0.961–1.195)	1.090 (0.977–1.216)	1.102 (0.990–1.227)
Lag 4	0.991 (0.891–1.102)	0.985 (0.885–1.095)	0.996 (0.895–1.108)	0.982 (0.882–1.094)	0.994 (0.893–1.106)	0.986 (0.885–1.098)	0.986 (0.886–1.098)	0.997 (0.896–1.109)
Lag 5	0.789 (0.708–0.880)	0.792 (0.710–0.884)	0.791 (0.710–0.883)	0.791 (0.709–0.882)	0.799 (0.716–0.892)	0.792 (0.710–0.884)	0.795 (0.713–0.887)	0.785 (0.704–0.875)

Lag 01	1.235 (1.132–1.348)	1.210 (1.103–1.327)	1.249 (1.144–1.364)	1.258 (1.142–1.386)	1.213 (1.106–1.330)	1.242 (1.126–1.369)	1.256 (1.140–1.383)	1.320 (1.203–1.448)
Lag 02	1.144 (1.054–1.241)	1.111 (1.018–1.214)	1.161 (1.068–1.262)	1.153 (1.050–1.265)	1.120 (1.025–1.223)	1.133 (1.030–1.245)	1.155 (1.052–1.267)	1.210 (1.109–1.321)
Lag 03	1.062 (0.983–1.147)	1.024 (0.943–1.112)	1.074 (0.993–1.163)	1.049 (0.962–1.145)	1.032 (0.950–1.122)	1.031 (0.943–1.126)	1.052 (0.964–1.148)	1.101 (1.015–1.194)
Lag 04	1.052 (0.978–1.132)	1.019 (0.943–1.102)	1.063 (0.987–1.145)	1.039 (0.959–1.127)	1.027 (0.950–1.110)	1.025 (0.944–1.112)	1.042 (0.961–1.130)	1.082 (1.002–1.168)
Lag 05	1.003 (0.934–1.078)	0.970 (0.899–1.046)	1.012 (0.940–1.089)	0.985 (0.912–1.064)	0.978 (0.906–1.055)	0.969 (0.896–1.049)	0.990 (0.916–1.070)	1.020 (0.947–1.098)

CI, confidence interval; OR, odds ratio.

We additionally performed a dose-response analysis of the basic model, classifying AD days using the AD extinction coefficients. In the case of bronchial asthma, the ORs were also statistically significant for lag day 3 or lag day 4 (eTable 1). For respiratory diseases, the ORs at lag day 0, lag day 1, and lag day 2 were elevated (eTable 2).

We also carried out a sensitivity analysis in which we excluded visits within 4 weeks of the initial visit. This gave us larger adjusted ORs than the previous results (eTable 3). We also compared whole-year data with those obtained between March and May; the ORs were lower with the whole-year data (eTable 4).

## DISCUSSION

In this study, we found that exposure to AD led to increased emergency department visits by children. The association might vary between school and preschool children, though we could not detect a statistical interaction due to the small sample size. The association between AD exposure and increased emergency department visits due to bronchial asthma was might be stronger in school children than in preschool children, and the effect of exposure was manifested in a delayed fashion.

AD particles contain rock-forming minerals, such as quartz and feldspar, and argillites, such as mica, kaolinite, and green mudstones. However, they also have sulfur oxidants, nitrogen oxidants, ammonium, and microorganisms attached to them, and these may have adverse health effects, including allergic reactions^27^ and inflammation of the bronchi.^28^ In Taiwan, Yu et al reported that the relative rate of clinic visits by children for respiratory diseases decreased significantly (by 0.74 to 0.99 times) in most districts during AD-storm periods, while it rose by 1.01 to 1.11 times in more than half of the districts during the period following AD storm, especially in school children.^9^ In contrast, Ueda et al reported no significant association between AD exposure and asthma hospitalization among children in Fukuoka, Japan.^15^

A possible explanation for our findings of a stronger association between AD exposure and emergency department visits due to bronchial asthma in school children than preschoolers is that the former participate in more outdoor activities and subsequently have greater exposure. Bronchial asthma is also more easily diagnosed in school children, and it is sometimes indistinguishable from other respiratory diseases in preschool children, such as bronchiolitis. Furthermore, preschool children with bronchial asthma might be under more careful management by their parents than school children.

Our study also revealed a delayed effect of AD exposure, with the strongest effect observed 3 or 4 days after exposure. This is consistent with some earlier findings.^29^^,^^30^ Yang et al reported that the effects of dust storms on admissions for asthma were most prominent 2 days after the event; however, this result was not statistically significant.^29^ Meng et al reported that dust events were significantly associated with total respiratory hospitalizations 3 days later in both boys and girls.^30^ This delayed effect may be due to physiological reasons; the respiratory function of school children is more mature than that of preschool children, so the older children can tolerate asthmatic symptoms to a greater extent. Another explanation may be delayed emergency department visits, as school children may not visit on the day when they notice the symptoms because they do not want to miss school. Moreover, cough or wheeze and dyspnea with bronchial asthma are likely to worsen at night time.

As for the association between AD exposure and respiratory diseases, this was stronger in preschool children than in school children, and the effects were immediate. Although this association has been reported previously, the findings have been inconsistent in terms of susceptibility by age group and lag period. A study in Taipei showed that the rate of clinic visits for respiratory diseases during the weeks following AD events increased by 2.54% in preschool children and 5.03% in school children.^8^ In another study, post-AD days 1 through 4 saw significantly higher mean numbers of pneumonia admissions than non-AD days.^11^ Yu et al reported a significantly increased risk of respiratory diseases in the week after AD, especially in school children. The largest percentage change in preschool children was at lag day 2 and lag day 3 (2.12% and 2.19%, respectively), and the largest change in school children was at lag day 3 (3.17%).^10^

The stronger association between AD exposure and respiratory diseases in preschool children than in school children observed in our study may be explained by the under-developed respiratory system in preschool children and difficulty in making differential diagnosis of wheezing illnesses, including bronchiolitis and other respiratory diseases, as well as bronchial asthma.

One of the strengths of the present study was the use of LIDAR to assess AD exposure. In Japan, AD days are generally assessed on the basis of visual observations by the Japan Meteorological Agency. These observations are done every hour, and an AD day is recorded when AD is observed for at least 1 hour on that day.^31^ In contrast, LIDAR measurements allow the quantitative evaluation of the level of AD particles as a dust extinction coefficient.^18^ However, no cutoff point has been established to distinguish between AD days and non-AD days. Kanatani et al used a 24-hour average dust extinction coefficient of 0.1/km or more to define mild AD days.^14^ Ueda et al went further to define mild AD (dust extinction coefficient ≤0.066/km), moderate AD (0.066/km–0.105/km), and heavy AD days (>0.105/km).^26^ Establishing a proper definition of AD days is warranted in the future.

The high regional representativeness of our study is another strength. The NMPEM Center is the only hospital in the study area that children can visit during the night, and approximately 90% of patients who access this center live in the covered area. However, we could not obtain patients’ residential address because of laws governing the protection of personal information.

Time-stratified case-crossover analysis obviates the need to consider potential confounding by sex, age, and daily habits, which do not change over short periods, and also controls for the effects of long-term trends, seasonality, and the day of the week. However, a potential limitation of our study is that inflammation of the bronchi may continue for several days to several weeks after exposure, and inflammation makes the bronchi more vulnerable to environmental changes; therefore, those children with bronchial inflammation are more likely to revisit medical centers. To adjust for this, we repeated our analyses after excluding visits within 4 weeks of the initial visit and found that the results were substantially unchanged. In the sensitivity analysis, we compared the ORs in the whole year with those obtained between March and May, and found that the former was lower than the latter. Because most of the AD comes in the spring, we thought that the addition of seasons other than spring into the analysis would only attenuate the association between AD and outcomes.

Another potential limitation is a misclassification of exposure. LIDAR cannot measure the vertical distribution of AD any lower than 120 meters above the ground, so we cannot discount the possibility that the concentrations of particles our subjects actually inhaled were different from those measured. Nor did we obtain information on the patients’ daily activities, so we could not take these into consideration. As for the horizontal distribution of AD, the LIDAR we used was located in Omura City, which is approximately 30 km from the center of Nagasaki City. However, we believe that any resulting misclassification would have been non-differential and would have made the observed association between AD exposure and outcomes weaker than the true association. Misclassification of the outcomes is also possible. Had the emergency physicians known that AD was on its way, it could have led to an information bias. However, if the elevated ORs were due only to such bias, the results would have been similar between preschool and school children, which was not the case. Another possible limitation we should mention is that, although the emergency medical center is open from 20:00 until 06:00, we did not obtain the exact times of patients’ visits. This could also have led to outcome misclassification, but because any such misclassification was independent of exposure, it would have been non-differential. In addition, when comparing our results with those from other countries, we have to consider the differences in diameter and the chemical composition of AD and in the attached metals.^28^

### Conclusions

Our study showed that exposure to AD was associated with increased emergency department visits due to bronchial asthma in school children and with increased visits due to respiratory diseases in preschool children.

## ONLINE ONLY MATERIALS

eTable 1. Association between AD and emergency department visits due to bronchial asthma by categories of the AD extinction coefficients.

eTable 2. Association between AD and emergency department visits due to respiratory diseases by categories of the AD extinction coefficients.

eTable 3. Association between AD and emergency department visits by bronchial asthma among school children; all subjects vs subjects excluding those revisiting within 4 weeks.

eTable 4. Association between AD and emergency department visits by bronchial asthma among school children; comparison March–May vs whole year.

Abstract in Japanese.
