# Supplementary material for: Asian Dust and Pediatric Emergency Department Visits Due to Bronchial Asthma and Respiratory Diseases in Nagasaki, Japan
Source: J Epidemiol. 2016 Nov 5;26(11):593–601. doi: 10.2188/jea.JE20150309 (PMC5083323; doi:10.2188/jea.JE20150309)
Supplement: eTable 1. [file je-26-593-s001.pdf]

e Table 1. Association between Asian dust and emergency department visits due to bronchial asthma by categories of Asian dust extinction coefficient

		All Children (0-15 years old)	Preschool Children (0-5 years old)	School Children (6-15 years old)
Category	Lag	OR[a] (95% CI)	OR[a] (95% CI)	OR[a] (95% CI)
Non-Asian dust days Moderate Asian dust days Heavy Asian dust days	Lag 0	1.051 (0.930-1.187)	1.052 (0.911-1.215)	1.033 (0.818-1.306)
	Lag 1	1.043 (0.926-1.173)	1.051 (0.916-1.206)	1.009 (0.800-1.272)
	Lag 2	1.109 (0.989-1.243)	1.063 (0.928-1.217)	1.224 (0.987-1.519)
	Lag 3	1.120 (0.996-1.260)	1.059 (0.921-1.218)	1.293 (1.038-1.611)
	Lag 4	1.220 (1.084-1.373)	1.234 (1.073-1.420)	1.175 (0.943-1.464)
	Lag 5	0.909 (0.797-1.035)	0.856 (0.733-0.999)	1.072 (0.838-1.373)

Non-Asian dust days (Asian dust extinction coefficient <0.047/km), Moderate Asian dust days (0.047/km-0.065/km)
Heavy Asian dust days (≥0.066/km)

[a] Odds ratios (ORs) and 95% confidence intervals (CIs) for one unit increase in categories were estimated in basic models (adjusted by temperature and humidity)
