# Supplementary material for: Asian Dust and Pediatric Emergency Department Visits Due to Bronchial Asthma and Respiratory Diseases in Nagasaki, Japan
Source: J Epidemiol. 2016 Nov 5;26(11):593–601. doi: 10.2188/jea.JE20150309 (PMC5083323; doi:10.2188/jea.JE20150309)
Supplement: eTable 2. [file je-26-593-s002.pdf]

e Table 2. Association between Asian dust and emergency department visits due to respiratory diseases by categories of Asian dust extinction coefficient

		All Children (0-15 years old)	Preschool Children (0-5 years old)	School Children (6-15 years old)
Category	Lag	OR[a] (95% CI)	OR[a] (95% CI)	OR[a] (95% CI)
Non-Asian dust days<br>Moderate Asian dust days<br>Heavy Asian dust days	Lag 0	1.119 (1.070-1.169)	1.123 (1.067-1.181)	1.106 (1.011-1.209)
	Lag 1	1.120 (1.073-1.169)	1.120 (1.065-1.176)	1.120 (1.029-1.220)
	Lag 2	1.064 (1.017-1.113)	1.064 (1.010-1.122)	1.061 (0.969-1.161)
	Lag 3	0.990 (0.945-1.038)	0.964 (0.913-1.018)	1.070 (0.975-1.173)
	Lag 4	0.970 (0.925-1.017)	0.946 (0.895-1.000)	1.041 (0.950-1.141)
	Lag 5	0.913 (0.870-0.959)	0.905 (0.855-0.957)	0.939 (0.853-1.033)

Non-Asian dust days (Asian dust extinction coefficient <0.047/km), Moderate Asian dust days (0.047/km-0.065/km)
Heavy Asian dust days (≥0.066/km)

[a] Odds ratios (ORs) and 95% confidence intervals (CIs) for one unit increase in categories were estimated in basic models (adjusted by temperature and humidity)
