# Supplementary material for: Asian Dust and Pediatric Emergency Department Visits Due to Bronchial Asthma and Respiratory Diseases in Nagasaki, Japan
Source: J Epidemiol. 2016 Nov 5;26(11):593–601. doi: 10.2188/jea.JE20150309 (PMC5083323; doi:10.2188/jea.JE20150309)
Supplement: eTable 3. [file je-26-593-s003.pdf]

e Table 3. Association between Asian dust and emergency department visits for bronchial asthma among school children; all subjects vs. subjects excluding those revisiting within 4 weeks

		All subjects	Subjects except revisiting cases within 4 weeks
Exposure	Lag	OR (95% CI)	OR (95% CI)
Asian dust (LIDAR)	Lag 0	1.291 (0.850-1.961)	1.401 (0.918-2.138)
	Lag 1	1.599 (1.032-2.478)	1.848 (1.176-2.904)
	Lag 2	1.394 (0.891-2.182)	1.626 (1.025-2.577)
	Lag 3	1.787 (1.174-2.721)	1.897 (1.234-2.916)
	Lag 4	1.807 (1.163-2.809)	1.848 (1.166-2.930)
	Lag 5	0.894 (0.550-1.453)	0.858 (0.512-1.438)
	Lag 01	1.329 (0.915-1.930)	1.497 (1.022-2.194)
	Lag 02	1.136 (0.794-1.625)	1.279 (0.886-1.845)
	Lag 03	1.173 (0.840-1.639)	1.273 (0.903-1.793)
	Lag 04	1.131 (0.822-1.557)	1.223 (0.882-1.696)
	Lag 05	1.020 (0.739-1.408)	1.108 (0.795-1.154)

CI, confidence interval; LIDAR, light detection and ranging; OR, odds ratio.
