# Supplementary material for: Asian Dust and Pediatric Emergency Department Visits Due to Bronchial Asthma and Respiratory Diseases in Nagasaki, Japan
Source: J Epidemiol. 2016 Nov 5;26(11):593–601. doi: 10.2188/jea.JE20150309 (PMC5083323; doi:10.2188/jea.JE20150309)
Supplement: eTable 4. [file je-26-593-s004.pdf]

e Table 4. Association between Asian dust and emergency department visits for bronchial asthma among school children; comparison March-May vs. whole year

		March-May	Whole-year
Exposure	Lag	OR (95% CI)	OR (95% CI)
Asian dust (LIDAR)	Lag 0	1.291 (0.850-1.961)	1.323 (0.914-1.914)
	Lag 1	1.599 (1.032-2.478)	1.203 (0.825-1.753)
	Lag 2	1.394 (0.891-2.182)	1.287 (0.903-1.834)
	Lag 3	1.787 (1.174-2.721)	1.463 (1.035-2.070)
	Lag 4	1.807 (1.163-2.809)	1.218 (0.837-1.774)
	Lag 5	0.894 (0.550-1.453)	0.898 (0.594-1.356)
	Lag 01	1.329 (0.915-1.930)	1.188 (0.864-1.633)
	Lag 02	1.136 (0.794-1.625)	1.070 (0.804-1.424)
	Lag 03	1.173 (0.840-1.639)	1.026 (0.791-1.329)
	Lag 04	1.131 (0.822-1.557)	0.941 (0.737-1.203)
	Lag 05	1.020 (0.739-1.408)	0.898 (0.706-1.143)

CI, confidence interval; LIDAR, light detection and ranging; OR, odds ratio.
