# Supplementary material for: Asian Dust and Pediatric Emergency Department Visits Due to Bronchial Asthma and Respiratory Diseases in Nagasaki, Japan
Source: J Epidemiol. 2016 Nov 5;26(11):593–601. doi: 10.2188/jea.JE20150309 (PMC5083323; doi:10.2188/jea.JE20150309)
Supplement: Abstract in Japanese. [file je-26-593-s005.pdf]

長崎市における黄砂と気管支喘息や呼吸器疾患による小児救急受診との関連

中村　孝裕[1]、橋爪　真弘[2]、上田　佳代[3]、清水　厚[4]、竹内　文乃[5]、久保　達彦[6]、橋本邦生[7]、森内　浩幸[7]、小田嶋　博[8]、北島　翼[9]、田代　香澄[10]、冨増　邦夫[11]、西脇　祐司[1]

1 東邦大学医学部社会医学講座衛生学分野
2 長崎大学熱帯医学研究所小児感染症学分野
3 京都大学大学院工学研究科　都市環境工学専攻　環境衛生学講座
4 国立研究開発法人国立環境研究所地域環境研究センター
5 慶應義塾大学医学部衛生学公衆衛生学教室
6 産業医科大学医学部公衆衛生学
7 長崎大学大学院医歯薬学総合研究科　小児科学
8 国立病院機構福岡病院　小児科
9 長崎県上五島病院　小児科
10 独立行政法人地域医療機能推進機構諫早総合病院小児科
11 長崎市夜間急患センター

【背景と目的】黄砂曝露による小児の呼吸器疾患への影響は未だ十分に明らかでない。黄砂と小児気管支喘息やそれを含む呼吸器疾患による救急外来受診との関連について検討を行った。
【方法】長崎市夜間急患センターを受診した小児の匿名化データ(2010 年 3 月～2013 年 9 月)を用いた。黄砂曝露の定義は LIDAR（Light Detection and Ranging）観測のデータに基づいて行い黄砂と救急受診の関連は Time Stratified ケースクロスオーバー解析を行い関連の強さはオッズ比と 95%信頼区間(CI)により示した。主たる解析は黄砂が多く飛散する 3 月から 5 月に限定して行った。
【結果】対象期間の救急受診は気管支喘息 756 件とそれを含む呼吸器疾患 5,421 件で、黄砂日は 47 日であった。学童に関しては、非黄砂日に対して黄砂日から 3 日後と 4 日後の気管支喘息受診のオッズ比はそれぞれ 1.837 (95%CI; 1.212-2.786)、1.829 (95%CI; 1.179-2.806)であった。一方で未就学児は黄砂日当日、翌日、2 日後にかけて関連がみられ、オッズ比はそれぞれ 1.244 (95%CI; 1.128-1.373)、1.314 (95%CI; 1.189-1.452)、1.273 (95%CI; 1.152-1.408)であった。これらの関連は気象因子や他の大気汚染物質で調整後も同様に統計学的に有意であった。
【結論】黄砂曝露により、気管支喘息や呼吸器疾患による小児救急受診が増加することが示唆された。
キーワード：黄砂、救急受診、気管支喘息、呼吸器疾患、小児
